# Severe acute respiratory syndrome coronavirus-2 (SARS-CoV-2) infection: let the virus be its own demise

**DOI:** 10.2217/fvl-2020-0068

**Published:** 2020-05-26

**Authors:** John Nemunaitis, Laura Stanbery, Neil Senzer

**Affiliations:** 1 ^1^Gradalis, Inc., Carrollton, TX 75006, USA; 2 ^2^Independent Researcher, Gradalis Advisor

**Keywords:** COVID-19, furin, GM-CSF, SARS-CoV-2, vigil

## Abstract

There has been a collaborative global effort to construct novel therapeutic and prophylactic approaches to SARS-CoV-2 management. Although vaccine development is crucial, acute management of newly infected patients, especially those with severe acute respiratory distress syndrome, is a priority. Herein we describe the rationale and potential of repurposing a dual plasmid, Vigil (pbi-shRNA^furin^-GM-CSF), now in Phase III cancer trials, for the treatment of and, in certain circumstances, enhancement of the immune response to SARS-CoV-2.

 Severe acute respiratory syndrome coronavirus-2 (SARS-CoV-2) is thought to have naturally evolved from two existing coronavirus strains (L and S) near Wuhan, China. Origin is presumed due to zoonotic transfer: the SARS-CoV-2 genome is 96.2% homologous to the bat RaTG13 coronavirus [1]. From 31 December 2019 to 4 April 2020, 1,133,373 confirmed coronavirus cases have been reported worldwide resulting in 60,375 deaths. Encouragingly, 235,999 patients have shown validated recovery. SARS-CoV-2 is emerging as a potentially greater morbidity and economic threat than the pandemic Spanish flu which infected 500 million people worldwide and resulted in 50 million deaths. The viral reproductive number (R_0_) of SARS CoV-2 is above two compared with 1.8 for the Spanish flu [2]. Although more highly infectious, SARS-CoV-2 has resulted in far less mortality and is related to primarily elderly patients with medical comorbidities. The current reported mortality rate seems to be holding at approximately 2% worldwide (although age and country related), however, considering shifting denominators, the case fatality rate may be lower; for example, 1.4% of those with laboratory-confirmed disease [3]. Since the pandemic of 1918, influenza has become endemic but with use of vaccines the infection rate has been reduced and the case fatality rate has dropped significantly to 0.1%. The SARS-CoV-2 pandemic, its rate of infectivity, related death, medical system overload and the consequent financial damage (due to disease, fear and quarantine) highlights the urgent need to develop new therapeutics as well as rapid response techniques to combat this and other novel pandemic virus outbreaks in the future.

In particular, patients who recover from SARS-CoV-2 show evidence of an effective immune response to clear the infection and stop viral shedding within approximately 3 weeks [1]. This is particularly important since the virus generally does not persist and viral clearance is achievable, although feasibility of reinfection is unknown given pulmonary site of infectivity and propagation. Efforts can be made to minimize the risk of acute respiratory distress syndrome (ARDS), by enhancing the existing immune response, and slowing the viral propagation process. We will discuss repurposing Vigil plasmid, which expresses GM-CSF and decreases furin expression. This approach inhibits multiples steps of viral propagation including viral entry, protein assembly and egress while GM-CSF confers cellular immune antiviral and lung protection activity. Pre-clinical and clinical data will be reviewed to highlight the rationale and evidence for combination approach.

## Brief history of recent human coronavirus infections/epidemics/pandemic

 Coronavirus was first identified in 1960 in a patient with an upper respiratory tract infection. The virus remained under the radar until 2002 when a patient with severe acute respiratory coronavirus (SARS-CoV) was identified in Guangdong, China. This virus rapidly spread to other hospital patients and staff, then spread globally to 37 countries. Eight hundred of the 8448 individuals diagnosed died [4]. After an initial delay, the measures that were taken limited the degree of dissemination and mortality in comparison to the 1918 pandemic.

The next coronavirus outbreak occurred in 2012 beginning in Saudi Arabia in a patient diagnosed with acute pneumonia, the cause of which was identified as Middle East respiratory syndrome coronavirus (MERS-CoV) [5]. 2500 cases of MERS-CoV infection were diagnosed in 24 countries of which 800 died before it's resolution in 2014 [6]. Thereafter another outbreak in humans occurred in 2015 in South Korea resulting in 186 cases and 36 deaths.

SARS-CoV-2, the virus responsible for the current coronavirus outbreak is genetically distinct from both the SARS-CoV and MERS-CoV viruses. The first patient was diagnosed in Wuhan, China and subsequently, the virus spread rapidly locally and then escaped regional containment. Over 80,000 cases and 3000 deaths were observed early on. Worldwide travel restrictions and social distancing measures have since been implemented in an attempt to slow the spread and thereby ease the global burden on healthcare workers and facilities. However, worldwide spread continues to occur although regional containment in China and South Korea has been reported but thus far no well-documented effective therapeutic approach has been found. Although discovery of rapid SARS-CoV-2 infection testing and antibody assessment diagnostics have facilitated identification of hot spot regions. Continued manufacturing of these tests will allow for rapid identification of individuals with SARS-CoV-2 or who have recovered and those who have antibody protection which will facilitate easing social-distancing measures.

## SARS-CoV-2 morbidity/mortality viral shedding & immune response

 Preliminary investigation of the first 191 patients in Jinyintan Hospital and Wuhan Pulmonary Hospital revealed that 54/191 (28%) died and 137/191 (72%) were able to be discharged [1]. Analysis of these first 191 patients determined that several factors significantly correlated with risk of death, including age >63 years old, high sequential organ failure assessment score (>1), high D dimer (>1 ng/ml), respiratory rate >24 breaths/min, lymphocyte count >.0.6 × 10^9^/l, elevated LDH (median 521 u/l) and elevated IL6 (median 11 μg/ml) as well as comorbidities, hypertension, diabetes, coronary artery disease and COPD [1]. Ninety three percent of the deaths were associated with ARDS and biopsy from one patient showing regions of pulmonary edema with hyaline membrane formation (early-phase ARDS) in one lung and desquamation of pneumocytes and hyaline membrane formation in the other (late-phase ARDS) [7]. There is suggestive evidence from the SARS-CoV epidemic that a dysregulated innate immune response and increase of pro-inflammatory cytokines (e.g., IL-1, IL-6 and IFNγ) may contribute to pulmonary pathology [8]. Notably, ARDS is also observed in chimeric antigen receptor CD-19 (CAR-T-CD19) therapy, which targets CD19 antigen and results in rapid induction of IL-6. Given that IL-6 is elevated to greater degree in patients who died from SARS-CoV-2 infection compared with healthy controls, tocilizumab, a monoclonal antibody targeted to IL-6, and used to manage ARDS associated with CAR-T therapy, may be a therapeutic component for those patients with elevated IL-6 [1,9].

Nasopharyngeal swabs from 79 patients at the First Affiliated Hospital, Nanchang University were obtained to serially assess viral dynamics via PCR-RT [10]. Using the ΔCT method, estimates of viral load were significantly higher by a factor of 60× in severe compared with mild cases and 90% of the latter tested negative by day 10 post onset whereas all the severe cases tested positive beyond that demonstrating both a higher viral load and prolonged shedding time. Using IgM and IgG immunofluorescence (IFA) in 16 patients in Munich, no detectable neutralizing antibody (NA) was detected between days 3 and 6 [11]. NA was detected after 2 weeks with limited suggestion of correlation with clinical course. In a recent study of 173 documented (rRT-PCR) cases (161 with serial assessment) of COVID-19 admitted to Shenzhen Third People's Hospital (11 January–9 February 2020), 32 (18.5%) with severe and 141 (81.5%) with mild disease, seroconversion rates in those with serial assessment (ELISA) for IgM and IgG using double-antigen sandwich ELISA for total Ab was 100% (median day 11) [12].

## Severe acute respiratory syndrome coronavirus-2 (SARS-CoV-2) dependence on furin

 The β-coronavirus genus is the etiological agent responsible for viral acute respiratory syndromes; the pandemic sarbecoviruses, SARS-CoV and SARS-CoV-2, and merbecovirus, MERS-CoV [13]. SARS-CoV-2 is responsible for the current pandemic of COVID-19 and is distinct from other coronavirus strains. SARS-CoV-2 relies on S1/S2 cleavage at viral entry as compared with SARS-CoV [14,15]. Following the attachment of the receptor-binding domain (S1) to the ACE2-binding cellular site, the affinity of which is 10- to 20-fold higher than SARS-CoV [14], the S1 subunit is shed resulting in a stable and accessible fusion domain (S2) subunit [16]. SARS-CoV-2 utilizes the plasma membrane fusion pathway rather than the more immunogenic endosomal membrane fusion pathway, which is used by SARS-CoV. Amino acid sequence differences in the SARS-CoV-2 HR2 region enhances binding affinity between heptad repeat-1 (HR1) and HR2 thereby accelerating viral membrane fusion [17]. The presence of a unique furin cleavage site (RRAR) at the S1/S2 boundary and the furin-like S2′ site located between fusion peptide (FP) and internal fusion peptide (IFP) sites on the S2 subunit may provide a gain-of-function allowing cleavage during viral egress thereby directly or indirectly contributing to increased replication rate, transmission and disease severity [15]. Note that proteolytic cleavage of the S glycoprotein can determine whether the virus can cross species, e.g. from bat. While structurally similar to SARS-CoV-2, the RaTG13/2013 virus lacks a unique peptide PRRA insertion region at the S1/S2 boundary [18]. Further, the S glycoprotein from a MERS-like coronavirus isolated from Ugandan bats can bind to human cells but cannot mediate virus entry unless incubated with trypsin prior to transduction allowing S glycoprotein cleavage and virus entry [19]. These observations suggest that cleavage of the S glycoprotein may be a prerequisite to coronavirus cross-species transmission. A recent publication from Nankai University (Tianjin, China) on SARS-CoV-2 reported that genome sequence analysis revealed a section of genes that was not present in SARS-CoV that had a cleavage site similar to HIV and Ebola which carry viral proteins necessary for fusogenic activity of viral species to the human cell membrane. To be activated, the viral fusogenic surface glycoprotein has to be cleaved by furin [20]. As mentioned, viruses contain surface glycoproteins which when cleaved by furin or other proprotein convertases (PC) are activated and viral propagation is achieved (i.e., avian influenza, HIV, Ebola, Marburg and measles viruses) [21–23]. Another PC necessary for viral entry is the transmembrane serine protease TMPRSS2, which is known to contribute to efficient SARS-CoV cell entry and Hoffman *et al.*, has produced *in vitro* data showing that SARS-CoV-2 also uses TMPRSS2 priming [24]. However, further assessment of furin cleavage *in vivo* is appropriate given fusion-mediated cell entry of SARS-CoV-1 rather than SARS-CoV via endocytosis, the presence of a unique furin cleavage site (RRAR) at the S1/S2 boundary and the furin-like S2′ site in SARS-CoV-2 and the combination of cell membrane entry fusion and differences in the SARS-CoV-1 HR1 domain, which may contribute to the typical syncytium growth pattern in infected cells rarely reported in SARS-CoV [17]. Inhibition of furin may be a therapeutic approach that has efficacy in SARS-CoV-2 and other viruses that contain a furin cleavage domain. Another immunotherapeutic intervention would be to increase the pulmonary expression of GM-CSF, which, *in vivo*, redirects macrophages from an M1 state of activation to an M2 activation state and enhances expression of anti-inflammatory mediators and perhaps allow more time for patients to mount an effective immune response against SARS-CoV-2 [25].

In addition to interfering in viral dynamics, a therapy-targeting host proteases rather than a viral epitope could also reduce the development of vaccine resistance due to mutation of nonessential viral-targeted antigens. For both reasons, furin is an attractive therapeutic target. It is highly conserved and genomically unrelated to viral replicative functions and antigenic drift [26,27]. We do not know how effective vaccination will be with SARS-CoV-2 given the low titers of NA in patients with COVID-19 and antigenic drift characteristic of human host RNA viruses. Vaccination for influenza virus is only effective in 60% of individuals due to rapid antigenic evolution.

## Furin

 Furin, was first described in 1986 and is the product of the *fur* gene [28]. It is an evolutionarily conserved family member of the proprotein convertases which contain a subtilisin-like protease domain and was the first proprotein convertase (PC) to be identified in humans [29–31]. Furin is a type I transmembrane protein that is ubiquitously expressed in vertebrates and invertebrates [32]. It is localized to the Golgi and *trans*-Golgi network where it cleaves multiple proteins and is also located on the outer membrane where pathogens utilize it to cleave glycoproteins, a step essential for entry into host cells [15,21]. It can be secreted as a soluble, truncated active enzyme [21,33,34]. The correct folding of furins catalytic domain relies on the inhibitory function of the N-terminal 83-amino acid propeptide [35]. To gain its enzymatic activity, the inhibitory propeptide is removed during transport from the endoplasmic reticulum to the *trans*-Golgi network [36]. In order to be released into the extracellular space, the membrane localization is cleaved at the C-terminus [37]. Due to furin's ubiquitous expression and localization it is able to process a large amount and variety of proteins including growth factors, cytokines, hormones, adhesion proteins, collagens, membrane proteins, receptors as well as other classes [38]. Furin cleavage can also inactivate other proteins [39,40]. Its cleavage consensus sequence is Arg-Xaa-(Lys/Arg)-Arg↓-Xaa [41,42].

Many viral pathogens including, coronavirus, flavivirus, pneumovirus, avian influenza, influenza A and HIV, utilize furin-mediated membrane glycoprotein cleavage facilitate viral entry and, for certain viruses, egress from target host cells [29]. HIV-1 utilizes furin to cleave the viral membrane protein (Env) gp160 into gp120 and gp-41 prior to mature virion assembly. Conversely, flavivirus rely on furin cleavage after formation of packaged virions. SARS-CoV-2, as noted above, is cleaved at two sites, S1/S2 furin cleavage site (P**R**RA**R**↓S**V**) and a furin-like S2′ cleavage site (**KR**↓**SF**) [15].

## Viral response to furin protein inhibitors

 RNA viruses such a SARS-CoV-2 have several critical functions dependent upon protease activity. Consequently, modulation of protease activity may provide therapeutic function in SARS-CoV-2 in a variety of other RNA viruses. Furin is a particularly promising opportunity for therapeutic intervention. As previously described it cleaves and activates numerous mammalian, viral and bacterial substrates [38]. Becker *et al.* optimized preclinical therapeutic performance of several peptidomimetic furin inhibitors and demonstrated ‘*in vitro*’ significant inhibition of highly pathogenic H7N1 influenza virus propagation [43].

Although mechanisms have evolved enabling RNA viruses to invade host cells, host defense mechanisms have also evolved. Innate and adaptive immune responses have been shown to target viral antigens. Additionally, targets critical to viral entry, protein assembly and egress are also of high therapeutic value. These are ‘virus dependency factors’. Various host proteins such as IFI16 and SAMHD1 have been shown to inhibit both RNA and DNA viral gene expression and replication, respectively [44,45]. Furin is critical for viral membrane fusion, protein assembly and propagation, particularly as related to SARS-CoV-2.

Multiple furin inhibitors have been developed and tested *in vitro* and in animal models. Initial targets were peptide and protein inhibitors which target active sites and competitively inhibit binding sites. As example, two IFNχ-inducible GTPases, guanylate-binding proteins 2 and 5 (GBP2 and GBP5), with inhibitory furin activity have demonstrated cleavage inhibition of the HIV Env precursor gp160 and reduced HIV virion infectivity [28]. Control of furin expression with protease activated receptor 1 (PAR1), impacts downstream furin function and processing of human metapneumovirus F protein in HIV [46]. Associated neurocognitive disorders also provides evidence of resistance mechanisms that can occur while inhibiting spread of HIV-1 [47]. Another example, α-1 antitrypsin Portland (α1-PDX) inhibits both PC5K5 and furin. α1-PDX has been shown to inhibit processing of HIV-1 Env and measles virus F [48,49]. Moreover, peptides involving the cleavage site of influenza A virus hemagglutinin compete for furin activity [50,51]. Activation of MMP9 is also inhibited by autoinhibitory propeptide of furin [52,53]. These data support therapeutic development involving furin inhibition against SARS-CoV-2.

Interestingly, corneal damage in mice related to *Pseudomonas aeruginosa* has been shown to be reduced by non-D-arginine (D9R) and other furin inhibitors [54]. Nonpeptidic furin inhibitors have also demonstrated antifurin activity in the nanomolar dose range [55]. 2,5-dideoxystreptamine shows unusual furin inhibiting activity whereby a complex is formed with furin involving two molecules with separate functions, which interfere with the catalytic triad conformation and binding to an adjacent peptide stretch to inhibit furin activity [56].

Toxic effects related to furin inhibitors have not been observed outside of embryonic models. A study of furin-deficient mice demonstrated a critical role of furin during embryogenesis in which knock-out of the *fur* gene led to death by day 11 due to the failure of ventral closure and embryonic turning [57]. Therefore, furin inhibition should be limited to the non-pregnant population. Liver-specific interferon-inducible furin knock-out mice have not demonstrated adverse effects outside of embryogenesis implying that other proprotein convertases may compensate for furin deficiency given overlapping activity [58,59]. Targeting furin, a host enzyme, also avoids the emergence of resistance due to viral antigenic drift as described earlier as furin genome is highly conserved and maintains a stable genomic structure, while SARS-CoV-2 target sites undergo mutational changes throughout the viral life span and pandemic period [26]. Furin inhibitors also function as mentioned previously via knockdown at the RNA level [i.e., Regnase-1 (ZC3H12A), Roquin (RC3H1)] [60]. A concern, however, with modulation of Regnase-1 and Roquin is that both agents will most likely result in off-target effects as these products both degrade off target mRNA. The results outlined and safety profile support potential role of furin inhibitors within a pandemic and possibly even within the anti-terrorist government protection ‘tool box’.

## GM-CSF antiviral activity

 Similar to SARS-CoV-2, alveolar epithelial cells are the primary target of influenza virus (IV) and are the first site of entry and support for viral propagation and replication. Proinflammatory immune response is rapidly initiated toward viral cytopathogenic effect which leads to alveolar epithelial cell (AEC) apoptosis [61]. However, when infection persists and viral propagation continues leading to intensified inflammatory response, capillary and alveolar leakage occurs, followed by severe hypoxemia and eventually ARDS which requires hospitalized management, oxygen support and often ventilation assistance [61,62]. Clearance of the viral pathogens from the lung by immune effector cells and the initiation of epithelial repair processes including expansion of local epithelial progenitor cells to begin resealing of the epithelial layer are critical for medical recovery and prevention of hospitalization, oxygen and ventilation support in IV-induced lung injury. The majority of mortality in relation to SARS-CoV-2 infection has been related to ARDS leading to hospitalization and ventilation support which is testing our medical capacity [63,64]. However, the inflammatory immune response against the virus needs to be balanced between the elimination of virus and toxic effect of immune-mediated pulmonary injury in order to limit damage to the respiratory tract and alveolar cells which prevent ARDS [65]. Mononuclear effector cells (macrophages, dendritic cells, CD8^+^, neutrophils and lymphocytes) carry the bulk of the load in IV clearance and ‘balanced’ immune response against IV [64]. Similar activity demonstrated with IV is important for clearance of SARS-CoV-2. GM-CSF has been shown to promote proliferation, differentiation and immune activation of monocytes, granulocytes, macrophages [66,67]. GM-CSF in the lungs is mainly expressed by AEC type II cells [68] and is a first cytokine responder in protection of the lung environment, AEC survival and function, and is a positive prognostic factor in clearance of IV infection. Expressed GM-CSF in pulmonary secretions can potentially be used as an indication in bronchial lavage samples of early response and resistance thereby affecting medial need involving O2 support. Other cell types produce GM-CSF, but AECs have been shown to upregulate GM-CSF in the distal lung parenchyma upon IV infection, and then produce high levels of GM-CSF in the alveolar surrounding secretions [69]. AEC GM-CSF secretion with IV infection resolution appears to be further mediated via HGF/c-Met and TGF-α/EGFR signaling [70].

Relationship of GM-CSF to immune response activation against cancer and viral infection is well described [71–75]. GM-CSF also regulates the differentiation, proliferation and activation of alveolar macrophages [76,77]. *In vitro* studies indicate that GM-CSF causes rapid proliferation of alveolar type II epithelial cells thereby serving in repair and barrier protection of the respiratory epithelium at early stages of acute inflammation [68]. It is also known that GM-CSF expression from alveolar type II epithelial cells facilitates surfactant homeostasis further enhancing protection of viral induced pathology [76,78,79]. GM-CSF also enhances the antiviral responses of alveolar macrophages. Indeed, elevated levels of GM-CSF may elicit a biphasic M1↔M2 response pattern [80]. Although a number of studies show that GM-CSF and type I interferon act together to modulate macrophage polarization toward the M1 state of activation, recent *in vivo* studies conclude the opposite [80–82]. GM-CSF enhances viral clearance through expression of scavenger receptors, SR-A and MARCO [83–86]. These two receptors aid in viral clearance through activation of receptors TLR-3, TLR-9, NOD-2 and NALP-3 [87–89]. GM-CSF enhances mucosal immune responses and the effectiveness of DNA vaccines [90,91]. Recombinant human GM-CSF has been delivered to the lung and conferred resistance to IV infection [92,93]. Transgenic mice that constitutively expressed human GM-CSF exposed to IV, were able to mount and effective antiviral response that resulted in increased numbers of human alveolar macrophages [94]. Halstead *et al.*, also showed how GM-CSF overexpression after IV virus infection in a GM-CSF transgene mouse model prevents mortality [82]. Protective effects of GM-CSF against IV-A pneumonia have been seen in mice with constitutive and inducible GM-CSF expression models in alveolar type II epithelial cell transgenic mice with GM-/- and *GM +/+* pulmonary specific promoters (*SFTPC*, *SCGB1A1*), respectively [80]. This model was able to show GM-CSF enhancement of alveolar cell activity as indicated by increased expression of SP-R210 and CD11c expressive mononuclear cells. In mice lacking SR-A and MARCO, two receptors regulated by GM-CSF, MARCO was shown to increase expression of SP-R210 on alveolar macrophages and decrease resistance to IV. However, although continuous *SP-C-GM+/+* transgenic mice resisted early mortality from IV, concern was raised to continuous high GM-CSF exposure over prolonged time. Late assessment of lung tissue sections revealed the histological features of degenerative desquamative interstitial pneumonia at day 29. Degeneration of alveolar structure and large spaces containing desquamated cells characterized the lungs of high GM-CSF exposed mice. The results indicate that excessively high levels of GM-CSF impair appropriate tissue healing resulting in development of interstitial lung disease secondary to IV pneumonia and provide guidance for early large animal assessment and Phase I monitoring of patient safety. However, results support that the conditional GM-CSF expressive mice do well and have long-term survival advantage to IV infection and have significant advantage over untreated controls. Expression of GM-CSF either through transgenic or pulmonary delivery conferred survival advantage to influenza virus compared with WT mice that did not survive infection. When alveolar phagocytes were depleted, the protective effect also diminished suggesting that these cells are necessary to induce the innate immune response [93].

As described, infection with SARS-CoV-2 can progress to rapid induction of viral pneumonia and ARDS resulting in fatal outcome [95]. AECs play a critical role in orchestrating the pulmonary antiviral host response [96]. However, with early IV and SARS-CoV-2 infection AEC's release GM-CSF. GM-CSF heightens immune function of alveolar cells which leads to improved epithelial repair processes. During IV infection, AEC-derived GM-CSF also enhances a lung-protective mechanism. Similar results are seen with local rhGM-CSF application. This early use and/or enhancement of immune function and alveolar protection with elevated GM-CSF expression appears to overwhelmingly benefit clinical response. However, GM-CSF expression late in the inflammatory lung response is less well characterized. Although correspondence by Herold S *et al.* in using recombinant GM-CSF (leukine, Bayer HealthCare Pharmaceuticals, WA, USA) and an Aeroneb Solo nebulizer to administer leukine (125 μg/dose) demonstrated significant clinical benefit in four of six patients with ARDS related to infectious pneumonia (including two with H1N1 virus) [97]. Immune function enhancement was also shown in the leukine treated patients compared with untreated ARDS patients in analysis of pulmonary immune response, which is similar to preclinical evidence (*in vitro* and animal models) [69,93,98,99]. GM-CSF treated patients demonstrated alveolar cell protection, enhanced alveolar cell activity toward viral and other infectious clearance and shift to M1 response as assessed by increased alveolar CD80^+^ cells and CD206 drop. These results support enhancement of GM-CSF expression even late in pulmonary inflammatory response to viral infection may be of benefit which suggest therapy benefit in late stage ARDS patients. Safe administration of GM-CSF via inhalation therapy in 19 patients with autoimmune pulmonary alveolar proteinosis was also demonstrated to show benefit by Ohashi *et al.* [100]. Elevated IL-17 in bronchial alveolar lavage fluid was shown as a GM-CSF induced cytokine and may serve as a biomarker associated with benefit.

Sever-Chroneos *et al.* [80], found worsening IV infection and response in GM-CSF deficient mice was due to impaired IV clearance by macrophages. This work is supported by Berclaz *et al.* who demonstrated that the Fcγ receptor (FcγR)-mediated opsonophagocytosis of invaded pathogens by alveolar macrophages is related to GM-CSF [101]. T-cell-produced interferon γ (IFN γ) also effects alveolar macrophage FcγR expression which in turn stimulate production of IFN γ and other cytokines such as IL-18 and IL-12 supporting involvement of both innate and adaptive immunity turn on. Elevated alveolar GM-CSF level in transgenic mice also improves resistance of alveolar cells in association with IV infection [93]. GM-CSF has also been shown to be an important stimulator of CD8^+^ T lymphocytes and further enhances their role to activate DC priming in lymphoid tissue, thereby providing a positive feedback in further stimulation of CD8^+^ T cell expansion [102]. Greter *et al.* [103], also showed GM-CSF to be critically important for induction of CD8^+^ T-cell immunity. Chen *et al.* [104] also found GM-CSF to promote B-cell maturation and production of IV specific antibodies. During IV pneumonia, extensive additional *in vivo* data support the role of GM-CSF as a lung barrier-protectant and positive immune response factor [69,80,92,93]. AEC-expressed GM-CSF directly benefits the injured epithelium and is important in enhancing epithelial proliferation in the setting of hypoxic lung injury via repair of barrier function, reduction of capillary leak and return of tissue to homeostasis [68,105].

The data discussed above regarding targeting furin and increasing GM-CSF expression warrants further investigation to target SARS-CoV-2 infection. Vigil, which combines bifunctional shRNA targeting furin and incorporating a GM-CSF DNA sequence in a plasmid delivery vehicle (pbi-shRNA^furin^-GM-CSF) has been described as the most advanced anti-furin technology in clinical testing [28]. Vigil is an autologous tumor cell vaccine, with dual function that knocks down furin expression as seen by decreased expression of downstream proteins TGFβ1/2 and expresses GM-CSF [106]. It has demonstrated clinical success in several cancer populations but especially Ewing's sarcoma and ovarian cancer [107–110]. It has a demonstrated safety profile with no evidence of grade 3 product related toxicity effect following 1406 doses in 233 cancer patients. The potential efficacy and use of Vigil for COVID-19 is an example of the rational repurposing of drugs from indicated to nonprimary target disease alternatives. Such an approach could accelerate the clinical development process particularly urgent given the current COVID-19 pandemic.

## Anti-furin therapeutic: GM-CSF bi-shRNA^furin^ plasmid (VP)

**Figure 1.**
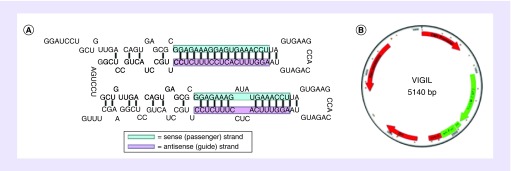
Vigil plasmid structure. **(A)** Furin bifunctional shRNA structure. **(B)** Schematic diagram of the Vigil plasmid. Vigil plasmid is 5140 bp, with a kanamycin cassette and CMV promoter. Vigil plasmid also contains the GM-CSF gene for expression and bifunctional furin sh-RNA. **(A)** Reproduced with permission from [111]. **(B)** Adapted with permission from [111].

VP constructed by Gradalis, Inc. (TX, USA), consists of two stem-loop structures with a miR-30a backbone [111]. The bi-shRNA^furin^ DNA as shown in Figure 1A uses a single targeted site to induce both mRNA cleavage and sequestration in P-bodies (translational silencing) and/or GW-bodies (repositories) [112]. By the use of this proprietary process, the encoding bi-shRNA can accommodate mature shRNA loaded onto more than one type of RNA induced silencing complex (RISC) [113]. Also, with the bi-shRNA^furin^ molecular design focusing on a single site potential toxic effects are reduced. Targeting of multiple sites increases chance for a ‘seed sequence’ being induced and leading to off-target effect that could result in increased clinical toxicity. Synthetic complementing and interconnecting oligonucleotides via DNA ligation were used to assemble the two stem-loop double stranded DNA sequences [114]. The 241 base pair DNA constructed with Bam HI sites at both ends was inserted into the Bam HI site of a prior clinically validated plasmid called TAG [115] in which we removed a TGFβ2 antisense DNA sequence and placed the bi-shRNA^furin^-GMCSF DNA sequence. Orientation of the inserted DNA was validated by the appropriate PCR primer pairs designed to screen for the shRNA insert and orientation. Safety profile defined with the prior TAG clinical therapeutic was used to support clinical advancement of VP in experimental cancer management testing under FDA guidance.Vigil is designed with the mammalian promoter cytomegalovirus [CMV] that drives the cassette. In between the GM-CSF gene (with a stop codon) and furin bi-shRNA there is a 2A ribosomal skip peptide followed by a rabbit poly-A tail. The picornaviral 2A sequence allows the production of two proteins from one open reading frame, by causing ribosomes to skip formation of a peptide bond at the junction of the 2A and downstream sequences [116]. Since we previously demonstrated the 2A linker to be effective for generating similar expression levels of GM-CSF and anti-TGFβ transcripts with the TAG vaccine and we observed robust activity in product release testing of therapeutic effector components expressed with this plasmid design along with clinical benefit and safety, we maintained the same design for VP. Transient expression of bi-shRNAfurin-GM-CSF plasmid and diluted expressive cell numbers in patients would not be expected to approach continuous toxic effect of transgenic models.

Gradalis has been clinically testing this plasmid since 2009 [111]. Aldevron (ND, USA) and Waisman (WI, USA) have participated in lot manufacturing. This plasmid, which consists of a bi-shRNA^furin^ DNA sequence and a GM-CSF DNA sequence (Figure 1b), has been validated for FDA registration trial based on significant cancer patient benefit for use of transfection (via electroporation) into an autologous tumor for vaccine (Vigil) construction in Ewing's sarcoma [107,117–121]. Additional benefit has also been suggested in a variety of other cancer types in Phase I and II testing, most notably ovarian cancer [71,109,122–124]. The current active lot of Vigil plasmid was manufactured in 2015 by Waisman. The plasmid concentration per vial is 2.2 mg/ml which provides 770 μg of plasmid. Yearly stability testing has passed all measures of evaluation under US FDA review. Recent double blind randomized control trial involving 25 nationally acclaimed sites unblinded and revealed OS advantage (HR: 0.417, p = 0.020) and RFS advantage (HR: 0.459, p = 0.007; stratified Cox's proportional Hazard Model) [125].

## Aerosolized therapeutics for viral pneumonia

Viral pneumonia, particularly in elderly or immune compromised patients, can be associated with devastating medical consequence [126]. Pulmonary delivery via aerosolized systems are simple, nonexpressive, noninvasive and allow for pain-free access of therapeutic and minimization of possible systemic side effects [127,128]. Aerosols have been shown to deliver plasmid DNA droplets with size ranging from 1 and 5 μm, which are able to disperse to the bronchial and alveolar epithelial cells. This enables pDNA entry and maximizes subsequent gene expression [129]. Rajapaksa *et al.* successfully demonstrated the use of a SAW liquid nebulization device for the generation of aerosolized pDNA with suitable size and stability characteristics to facilitate effective pulmonary delivery particularly for IV vaccination [130]. *In vivo* studies have shown successful pDNA delivery in both small and large animals. SAW nebulization used to deliver a plasmid vaccine demonstrated expression of protective anti-hemagglutinin (HA) antibodies. Anti-HA antibody titers detected were comparable to vaccination outcomes of other similar pDNA influenza vaccines not using a nebulizer [131]. These results support use of naked pDNA for effective delivery via pulmonary distribution while also demonstrating product stability and function. Following pDNA vaccination in rats, revealed higher serum hemagglutination inhibition (HAI) titers which were identified as protective according to WHO standards [132]. However, at this time, the SAW nebulizer approach has not demonstrated scale up capability for use in a pandemic event.

Aerosolized ribavirin however has demonstrated large volume capacity and adequate aerosolized delivery and clinical benefit including use in morbid condition patients. Ribavirin is indicated therapy for severe RSV infection in children. The conventional continuous treatment of 60 mg of ribavirin/ml for 18 h was found to be effective. Aerosolized ribavirin (administered 20 mg/ml for 2 h three times daily) has also been effective in cancer patients with RSV infection [133]. Ribavirin inhalation method at intermittent high doses (60 mg/ml) over the same schedule in immune suppressed children with RSF infection was also well tolerated. Moreover, results demonstrated similar improved clinical response compared with standard therapy. There was also less adverse exposure to healthcare workers [134,135]. Parainfluenza virus is associated with potentially serious complications in high morbidity patients (i.e., heart-lung transplant, allograft rejection, bronchiolitis obliterans [136]. Inhaled ribavirin in this population was associated with clinical improvement [137]. Aerosolized ribavirin (60 mg/ml) was also effective against IV-A and B infections in mice [138]. Recently, aerosolized ribavirin (100 mg/ml) was shown to be effective in mice infected with lethal IV-A H3N2 virus, and resulted in >0% survival when given early (within 24–48 h) after infection [139]. Aerosolized ribavirin treatment has been used with success against metapneumovirus pneumonia [140]. Moreover, in treatment of pneumotropic human adenovirus, aerosolized ribavirin demonstrated greater benefit over intravenous ribavirin likely related to the more robust drug concentration achieved in the alveoli with aerosolized product compared with intravenous ribavirin therapy. Additionally, the aerosolized delivery did not appear to lead to cytotoxic effect [141]. S-FLU immunization provides a broad cell-mediated immune response to conserved viral antigens. Data reveal that immunization with S-FLU–expressing H1 HA (H1 S-FLU) DNA reduces the viral load in lungs after aerosolized challenge with the closely matched pdmH1N1 virus strain [142]. The reduction of viral load was shown to be optimal using aerosol administration when compared with intravenous S-FLU. However, viral neutralizing Ab was not observed in S-FLU–immunized pigs, and the reduction of viral load in the H1 are group correlated with the presence of IFNg–producing CD8 or CD4/CD8 double-positive cells in the bronchoalveolar lavage suggest adequate product delivery. These data provide proof of principle that S-FLU DNA can be efficiently delivered by aerosol to a large animal, supporting possible use of a nebulizer device as a method of immunizing patients. Aerosolized delivery may be further optimized with use of lipid-DNA complexes [143]. Others have also shown successful aerosol delivery of measles vaccine in humans and/or exosome/viral delivery [144,145].

The challenge to this approach is how to introduce plasmid DNA into the lungs without loss or damage to the plasmid. Plasmid DNA is highly prone to shearing, therefore methods with low shear forces are necessary for effective delivery of the supercoiled DNA. Both nebulizers and dry powder inhalers use low amounts of shear forces. Nebulizers however use aerosol droplets to deliver particles into the lungs, which may not be an effective method to deliver plasmid DNA, as DNA degrades while in solution if not stored appropriately. Additionally, nebulizers limit the concentration of product that can be delivered due to solubility. Dry powder inhalers are not limited by solubility and plasmid DNA would not need to be stored in solution [146–148]. This method also reduces shear stress and thermal degradation which results in a high concentration of quality plasmid delivered directly into the lungs.

## Conclusion

Accumulating knowledge of intracellular viral processing, molecular biology, viral dynamics, host immune mechanisms and immunokinetics will allow for the development of tools and methods to protect lung function, delay or prevent ARDS, enhance anti-viral resistance and institute prophylactic measures. The unique role of furin and the demonstration of robust viral clearance in all patients who survive SARS-CoV-2 infection supply the rationale and support for repurposing Vigil for treatment of patients with COVID-19. Knockdown of furin with Vigil would target multiple steps of viral propagation, including viral, entry, protein assembly and egress. Expression of GM-CSF would provide further therapeutic benefit, enhancing the immune response and AEC protection. The data, limited as it is, showing no obvious correlation between seroconversion and viral clearance, gives additional support to a multifunctional therapeutic approach to COVID-19, in other words, combining inhibition of a protease critical to viral entry and cell to cell transmission with an immune response modulator. Vigil is already involved in FDA characterization with a known product safety profile. Further testing will be necessary, including *in vitro* activity assessment against SARS-CoV-2 and large animal safety.

## Future perspective

SARS-CoV-2 presents unique challenges for clinical management and infection containment. While vaccination is important to foster immunity and protect at-risk populations, finding relevant and effective therapeutics that could work across multiple viral pathogens will remain important. Logical drug repurposing and compound testing will be critical for rapid response to not only this pandemic, but future pandemics as well.

**Executive summary**

- SARS-CoV-2 relies on furin cleavage for multiple steps of the viral replication process, including viral entry, protein synthesis and viral egress.
- GM-CSF provides a lung protective effect, which may help to prevent ARDS and therefore allow natural immune clearance and antibody generation.
- Vigil plasmid is an example of logical drug repurposing and targets multiple viral propagation steps.
